# ‘Being an Outsider’ and ‘Being an Insider’: A Focused Ethnography of Family Members' Visitation Experiences in an Adult ICU

**DOI:** 10.1111/jan.70340

**Published:** 2025-11-03

**Authors:** Lian Zhu, Susanne Kean, Catherine Clarissa, Jennifer Tocher

**Affiliations:** ^1^ Nursing Studies, School of Health in Social Science The University of Edinburgh Edinburgh UK; ^2^ Scottish Collaboration for Public Health Research and Policy (SCPHRP) The University of Edinburgh Edinburgh UK

**Keywords:** family involvement, family visitation, focused ethnography, intensive care, nursing, space, visitation policy

## Abstract

**Aims:**

To explore how the restricted visitation policy impacts family members' visitation experiences and perceptions in an adult intensive care unit (ICU).

**Design:**

Focused ethnography.

**Methods:**

Data collection included 39 observation sessions (totalling 65.3 h), 19 semi‐structured interviews with family members, and document analysis of policies relevant to ICU visitation. Fieldwork was conducted in a general adult ICU at a tertiary hospital in China from April 2021 to December 2021. Data were analysed using reflexive thematic analysis.

**Results:**

Family visitation was represented by ‘being an outsider’ and ‘being an insider.’ ‘Being an outsider’ illustrates that the restricted visitation policy operated as a structural mechanism constructing the ICU as the staff's territory, positioning families as outsiders by limiting their access, information, and involvement in patient care. ‘Being an insider’ captures how family members constructed the waiting area as a socially meaningful family space where they reclaimed presence and formed a supportive community.

**Conclusion:**

Family visitation was shaped by the intersection of structural constraints, culturally embedded family roles, and relational dynamics among families. Restricted visitation policies reinforced family members’ powerlessness and limited their involvement.

**Implications for the Profession and/or Patient Care:**

The findings highlight the need to critically reconsider restricted visitation policies. Flexible, context‐sensitive visitation approaches that take into account cultural norms and family roles may better support family involvement in ICUs.

**Impact:**

The study contributes theoretically informed and culturally grounded insights into how the restricted visitation policies function as active structural constraints on family members' visitation experiences. It supports global efforts to develop inclusive, family‐centred ICU visitation practices that recognise the structural and relational needs of family members.

**Reporting Method:**

The Standards for Reporting Qualitative Research guidelines were followed.

**Patient or Public Contribution:**

No patient or public contribution.

## 1 Introduction

Family members have been increasingly recognised as integral to the quality of patient care and as recipients of care themselves (Hwang et al. 2025). This perspective has driven global efforts in intensive care units (ICUs) to enhance family involvement (Schwartz et al. 2022). Flexible visitation policies are widely recommended as they help support family needs, improve family satisfaction, reduce emotional distress, and strengthen trust in ICU staff (Mitchell and Aitken 2017). However, considerable variations in ICU visitation policies persist across settings, ranging from brief 30‐min daily visits to 24‐h open access (Junior et al. 2018). ICUs in some Western countries, such as the United Kingdom (Gibson et al. 2012), Australia (Bailey et al. 2022), and France (Garrouste‐Orgeas et al. 2016), have increasingly adopted flexible visitation models. Nonetheless, restricted visitation policies remain common globally (Dragoi et al. 2022), including in mainland China, where a once‐daily restricted visitation slot is standard practice (Huang et al. 2025). The impact of such restricted policies on family members' experiences requires deeper exploration to inform more humane and evidence‐based visitation policies.

## 2 Background

Visitation policies in adult ICUs have been debated for decades (Kirchhoff et al. 1985). Historically, restricted visitation was established as the default practice, informed by the ICU's origins as highly specialised spaces where healthcare professionals delivered intensive care with minimal interference (Hwang et al. 2025). Within this context, family members were often regarded as passive visitors, whose presence was perceived as disruptive, posing potential risks for infection, and as threats to patient privacy and safety (Ning and Cope 2020). However, increasing evidence has challenged these assumptions (Wu et al. 2022), highlighting the psychological and emotional benefits of family presence and recognising family members as potential partners in care.

The COVID‐19 pandemic renewed global attention to ICU visitation policies, as many ICUs enforced strict restrictions to prevent infection transmission (McPeake et al. 2023). These restrictions caused negative psychological outcomes among family members, particularly during total visitation bans in the early pandemic stages (Digby et al. 2023). Families reported emotional disconnection and heightened uncertainty due to their physical absence and lack of effective communication with ICU staff (Digby et al. 2023). These consequences underscore the need to understand the impact of restricted visitation policies, especially in contexts where such restrictions existed before and continue after the pandemic.

Beyond clinical significance, family visitation carries ethical implications (Khaleghparast et al. 2017). As patients are increasingly recognised as embedded within family systems, some scholars argue that family visitation should be treated as a right rather than a privilege (Lassi et al. 2025). Flexible visitation policies, despite varying in implementation across settings, share a common foundation in the principles of family‐centred care (Hwang et al. 2025), aiming to reduce structural barriers to family presence and facilitate family involvement in ICUs. Family members highly value opportunities for physical presence, perceiving it as a means to demonstrate care, maintain emotional connection, and acquire information by observing patients' conditions (Liu et al. 2025). Being at the bedside also allows family members to witness care practices that the patient is receiving, and continuous presence provides reassurance (Kynoch et al. 2021).

Family visitation is a culturally meaningful practice (Van Keer et al. 2015), shaped by local values and social norms (Al‐Mutair et al. 2014; Van Keer et al. 2015). Culturally embedded role obligations may influence how family members perceive and perform visitation (Kynoch et al. 2021). For example, Van Keer et al. (2015) found that ethnic minority families in a multi‐ethnic ICU in Belgium, characterised by extended family structures, often sought more frequent visits, as they viewed visiting patients as a social and moral duty. Similarly, Al‐Mutair et al. (2014) reported that Muslim families in Saudi Arabia expressed strong needs for hands‐on involvement in care, tied to religious and caregiving responsibilities. In these contexts, restricted visitation policies exacerbated family members' feelings of exclusion and emotional distress (Al‐Mutair et al. 2014).

In the Chinese context, cultural norms rooted in Confucian principles, particularly filial piety and collectivism, significantly shape family expectations and responsibilities (Gu and Li 2023). These principles emphasise intergenerational solidarity, reciprocity, and moral obligations to provide care for family members (Gu and Li 2023), which may intensify tensions between institutional visitation restrictions and culturally embedded expectations. Yet, limited research has explored how prolonged restricted visitation policies impact family members' experiences in Chinese ICUs. Exploring these experiences through a culturally sensitive lens is crucial for informing more family‐centred visitation policies.

## 3 The Study

The study aims to explore how the restricted visitation policy impacts family members' visitation experiences and perceptions in a Chinese adult ICU.

## 4 Methods

### 4.1 Design and Philosophical Assumptions

The study adopted a focused ethnographic approach, enabling the researchers to immerse themselves in participants' everyday activities and social worlds (Knoblauch 2005). Guided by social constructionism (Crotty 1998), this approach assumes that meanings are constructed through participants' social interactions and continuously negotiated within their social settings. Focused ethnography facilitated a contextual understanding of how the restricted visitation policy was experienced and perceived by family members in a Chinese adult ICU (Coffey 2018). This study is reported according to the Standards for Reporting Qualitative Research checklist (O'Brien et al. 2014).

### 4.2 Study Setting and Recruitment

This study was conducted in a mixed medical‐surgical adult ICU of a tertiary‐level hospital in mainland China. This setting was selected for its location in a county classified as a low‐risk area during the COVID‐19 pandemic, minimising infection risks, travel challenges, and access barriers. At the time of fieldwork, this 20‐bed ICU provided specialised care for approximately 350 critically ill patients annually.

**FIGURE 1:**
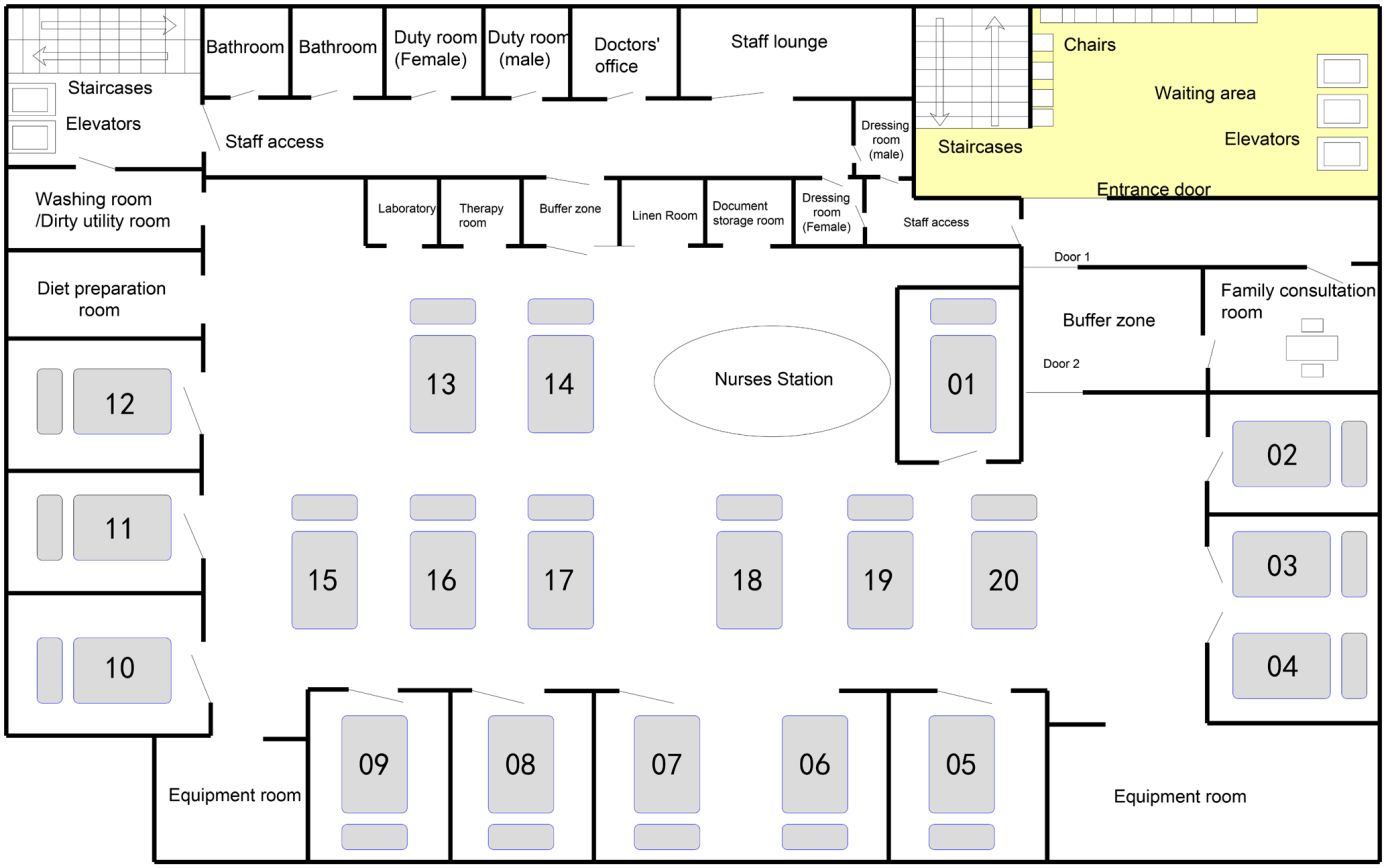
Layout of the studied ICU.

The ICU occupied an entire hospital floor, which was physically demarcated by a locked security door, clearly marking the boundary between the ICU and the waiting area. The layout of the ICU is presented in Figure 1. The waiting area outside the ICU was not a designated room, but rather an open communal space adjacent to the entrance door, furnished with two rows of steel chairs accommodating approximately 12 individuals. Posters in bold red font stating ‘No Visit’ and ‘No Unauthorised Access’ emphasised the exclusivity and restricted nature of the ICU. An information shelf in the corner provided informational leaflets and infection control brochures.

Following the first wave of the COVID‐19 pandemic in China (mid‐January to early April 2020), the ICU preserved its pre‐COVID‐19 30‐min visitation time but implemented additional infection prevention measures, including limiting visitors to one family member per patient and enhanced access procedures (Table 1). While acknowledging the immediate impact of these COVID‐19‐induced restrictions on family members' visitation experiences, this study focused on the longstanding practice of restricted visitation times and its impact on family members' visitation experiences and perceptions.

**TABLE 1:** ICU visitation policies before and during COVID‐19.

Visitation policy components	Before COVID‐19	During COVID‐19
Access procedures for visitors	PPE (face masks, gowns, shoe covers, hair covers), hand hygiene, no cold/flu symptoms	PPE (facial masks, gowns, shoe covers, hair covers), hand hygiene, no cold/flu symptoms, temperature checks, COVID‐19 negative test result, health QR code, personal information collection (home address, phone number, travel history, etc.)
Number of visitors	Three visitors (one at a time)	One visitor
Visitation time	30 min (15.00–15.30)	30 min (15.00–15.30)

*Note:* ‘Before COVID‐19’ refers to the period prior to the enactment of the first nationwide guideline for COVID‐19 Infection Prevention and Control within Acute Healthcare Facilities by the National Health Commission on 22nd January 2020. ‘During COVID‐19’ refers to the period of data collection for this study, from April 2021 to December 2021.

A purposive sampling strategy was employed to ensure diversity in age, gender, relationship to the patient, and admission diagnosis, enabling the collection of rich data reflecting a range of visitation experiences of family members. Posters were placed in the waiting area and the family consultation room inside the ICU to inform family members of the study. One ICU nurse who was responsible for admissions and discharge registration, along with two physicians, helped identify eligible participants. They helped inform potential participants about the study and referred interested individuals to the first author. The first author scheduled in‐person meetings to explain the study details in lay terms and provide study information sheets.

### 4.3 Inclusion and Exclusion Criteria

Family members were eligible if they: (1) were aged 18 years or older with continuing relationships with the ICU patient; (2) had visitation experiences, defined broadly to include both physical presence in the ICU and relevant activities such as waiting outside the ICU; (3) had a family member who had been admitted to the ICU for at least 24 h, to ensure sufficient time to reflect on their experiences.

Family members of end‐of‐life patients were excluded, as their unique visitation experiences were beyond the scope of this study.

### 4.4 Data Collection

The first author, a female Chinese nurse with a background in intensive care nursing and qualitative research training, conducted ethnographic fieldwork from April 2021 to December 2021 as part of her PhD study. Multiple data collection methods, including participant observation, document examination, and semi‐structured interviews, were employed to comprehensively explore family visitation and enhance rigour through triangulation (Coffey 2018).

Participant observation began the data collection, allowing the researcher to become familiar with the ICU context and the everyday practices surrounding family visitation. Observations took place both inside the ICU during the 30‐min visiting period and in the waiting area outside the locked ICU entrance. The researcher adopted the role of a participant observer, maintaining a primarily observational stance and engaging in informal conversations only when approached or to clarify observed events. In the waiting area, observations focused on the physical environment of the space, patterns of family behaviours, and interactions within and between families. Inside the ICU, the emphasis was on family members' bedside behaviours, their verbal and non‐verbal interactions with patients and ICU staff. Each participant was observed at least once in both spaces. The researcher positioned herself at a respectful distance to minimise disruption to family dynamics. Observation sessions lasted between 45 min and four hours, typically one to two hours. Fieldnotes recorded detailed descriptions of settings, interactions and behaviours of participants, alongside the researcher's reflexive memos (Hammersley and Atkinson 2019).

Document analysis involved two types of materials. First, policy‐related documents included the ICU's visitation guidelines and local/national COVID‐19 infection control policies. Second, everyday documents included family information leaflets, visitor passes, visitor logs, and informational posters displayed at the ICU entrance and waiting area. The analysis focused on the documents' practical use and their role in structuring family visitation, rather than content alone. These documents provided contextual depth to observational and interview data.

For semi‐structured individual interviews, an interview guide was developed based on research questions and refined throughout fieldwork (Supporting Information File S1). Follow‐up questions were tailored to participants' responses to probe deeper insights. Interview times and locations were negotiated with participants to accommodate their preferences and convenience. Interviews were primarily conducted in a private office near the ICU to ensure privacy and audio‐recording quality. Three interviews occurred in the waiting area, at participants' requests to remain physically close to the ICU; these were scheduled in the evening, when the space was quieter, to reduce interruptions and maintain confidentiality. All interviews were conducted in Mandarin and audio‐recorded with consent, lasting between 30 and 75 min.

Fieldwork concluded when the data collected were considered sufficiently rich to address the research questions. Ongoing reflexivity and constant comparison across data enabled the identification of recurring patterns. Final rounds of observations and interviews were used to confirm the preliminary interpretations, indicating the sufficiency of the data.

### 4.5 Data Analysis

Data collection and analysis occurred simultaneously, with systematic coding undertaken after leaving the field. Fieldnotes, documents, and interview transcripts, originally in Chinese, were managed using NVivo 13. Reflexive thematic analysis by Braun and Clarke (2022) was employed for its theoretical flexibility and alignment with qualitative paradigms.

Initial coding was conducted in Chinese to preserve cultural and linguistic nuances. The first author translated the codes and quotations into English, which were reviewed by two bilingual colleagues with backgrounds in psychology and nursing to ensure accuracy and clarity. Translated codes and quotations were regularly discussed with co‐authors to refine the analysis.

The analysis was iterative and recurrent, combining inductive and abductive reasoning. We inductively identified patterns in the data and generated codes and themes while abductively engaging with theoretical concepts of outsiders and insiders (Merton 1972) to provide deeper explanatory insights.

#### 4.5.1 Theoretical Concepts

The analysis was informed by Merton's (1972) structural conception of outsiders and insiders. Merton (1972) conceptualised these two statuses as dynamic social categories, continuously negotiated through interactions and embedded within specific social structures. This conception was applied alongside a spatial perspective (Delaney 2005), revealing how space is socially constructed, which in turn shapes social positions and relationships. This lens provides a nuanced interpretation of the impact of the restricted visitation policy on family visitation. Family members were positioned as outsiders in relation to the staff‐controlled ICU space, where access to the patient, information, and involvement were restricted, yet they simultaneously constructed insider status within the socially meaningful waiting area through presence, support, and interaction with their own and other families.

### 4.6 Ethical Considerations

Ethical approval for this study was obtained from the Research Ethics Committee of the University of Edinburgh (Reference No NURS059). Institutional approval was granted by the hospital and ICU through an official permission letter prior to the fieldwork. Written informed consents were obtained from all participants before interviews and observations. Pseudonyms were assigned to all participants to ensure confidentiality and anonymity (Table 2).

**TABLE 2:** Summary of participants.

Participant code	Pseudonym	Age	Gender	Data	Relationship to the patient	ICU admission diagnoses
P01	Anna	56	Female	PO/I	Mother	Toxicological
P02	Cathy	31	Female	PO/I	Daughter‐in‐law	Neurological
P03	Dave	47	Male	PO/I	Brother	
P04	Georgia	57	Female	PO	Wife	
P05	Albert	32	Male	PO	Son	
P06	Jasper	21	Male	PO	Son	Neurological
P07	Helen	46	Female	PO	Wife	
P08	Daniel	53	Male	PO	Son	Trauma‐related
P09	Rita	23	Female	PO	Daughter	Toxicological
P10	Janet	30	Female	PO/I	Daughter	Neurological
P11	Ken	46	Male	PO/I	Senior son	
P12	Leo	40	Male	PO/I	Younger son	
P13	Kelly	68	Female	PO/I	Wife	Trauma‐related
P14	Wade	40	Male	PO/I	Son	Neurological
P15	Martin	48	Male	PO/I	Father	Post‐surgical
P16	Ruby	46	Female	PO	Mother	
P17	Nick	29	Male	PO/I	Son	Post‐surgical
P18	Ben	79	Male	PO	Husband	Trauma‐related
P19	Paul	49	Male	PO/I	Senior son	
P20	Harley	42	Male	PO	Son‐in‐law	
P21	Grace	23	Female	PO	Granddaughter	
P22	Laura	37	Female	PO/I	Wife	Neurological
P23	Celia	40	Female	PO	Sister	
P24	Maggie	36	Female	PO/I	Daughter	Neurological
P25	Scott	48	Male	PO/I	Husband	Trauma‐related
P26	Simon	20	Male	PO/I	Son	
P27	Kevin	27	Male	PO	Son	Trauma‐related
P28	Nelson	31	Male	PO	Nephew	
P29	Oliver	35	Male	PO/I	Senior son	Respiratory‐related
P30	Amanda	48	Female	PO	Senior daughter	Trauma‐related
P31	Cherry	45	Female	PO/I	Younger daughter	
P32	Phil	41	Male	PO/I	Son	Sepsis and Infection‐related
P33	Bonnie	38	Female	PO	Daughter	Trauma‐related
P34	Jim	28	Male	PO/I	Husband	Neurological
P35	Peter	65	Male	PO	Husband	Neurological
P36	Cliff	39	Male	PO	Younger son	Cardiac

*Note:* PO: Participant Observation, I: Interview.

### 4.7 Rigour and Reflexivity

Reflexivity was a key strategy for ensuring the trustworthiness of this study (Hammersley and Atkinson 2019). The first author, who possessed a dual role as an ICU nurse and qualitative researcher, systematically documented reflexive journaling to critically examine the impact of her positionality on data collection, interpretation, and the framing of findings. Her clinical background enabled sensitive engagement with family member participants, particularly in managing emotionally charged interviews and observations, facilitating rapport and ethical handling of participants' responses. As a Chinese researcher, she also shared linguistic and cultural knowledge with participants, offering an emic perspective on the familial roles, norms, and unspoken expectations that shaped family visitation experiences. To avoid imposing assumed cultural explanations, the first author maintained analytical memos to document research decisions made throughout the fieldwork and analysis. The qualitative researcher's identity, however, offered an etic perspective, maintaining critical distance and analytical sensitivity. Regular discussions with co‐authors also helped challenge assumptions, critically interrogate interpretations, and enhance confirmability.

The trustworthiness of the research was supported by the robust research design. The triangulation of data sources and methods enhanced credibility and dependability by incorporating multiple perspectives on family visitation (Coffey 2018). Thick description, including detailed contextual information about the setting and participants, was employed to support the transferability of findings (Shenton 2004).

## 5 Findings

Data collection included 39 observation sessions (65.3 h), 19 interviews, and document analysis. A total of 36 family members participated, with 19 participating in both observations and interviews and 17 in observations only. Participants were aged 20–78 years and represented diverse familial relationships, primarily including adult children (47%), spouses (17%), siblings, in‐laws (14%), and other family members. ICU admission diagnoses of patients varied, predominantly neurological (36.4%) and trauma‐related (31.8%).

Two main themes represented family members' visitation experiences: ‘Being an outsider’ and ‘Being an insider’ (Table 3). ‘Being an outsider’ refers to family members' experiences of structural exclusion imposed by the restricted visitation policy. The physical and symbolic boundaries that limited access to patients, information, and involvement in care positioned family members as outsiders to the staff‐controlled ICU space.

**TABLE 3:** Themes and subthemes.

Themes	Subthemes
Being an outsider	Restricted access to the patient
	Being kept in the dark
	Exclusion from patient care
Being an insider	Receiving support from family members
	Connecting with other patients' families

In contrast, ‘being an insider’ captures how family members navigated this exclusion by reclaiming the waiting area outside the ICU. They constructed insider status in the waiting area by transforming it into their own territory where they reclaimed presence, formed supportive relationships, and exchanged emotional and informational resources.

### 5.1 Being an Outsider

#### 5.1.1 Restricted Access to the Patient

From the outset of ICU admission, family members were clearly informed about the unit's visitation policy through verbal instructions from nurses, information leaflets, and visible signs posted on the waiting area walls. This knowledge shaped their understanding and acceptance of visitation restrictions, as Cathy explained:

Only one person is allowed to visit, and there are 30 min of visitation time daily. Each family member who wants to visit inside must have a negative test result. (P02)

Family members respected the ICU as the staff's territory, acknowledging the necessity of visitation restrictions as infection control practices, particularly under the COVID‐19 pandemic, to reduce infection risks and protect their vulnerable family members. Anna described:

Sometimes, when the entrance door wasn't closed, some family members would sneak inside to catch a glance, but they were driven away immediately [laughs]. It's not even allowed to stand near that door […] no matter who saw us standing at the inside door, they asked us to leave. (P01)

However, the restricted access caused tension between family members' desire to be present and their enforced separation. The closed entrance door functioned as both a physical and symbolic barrier, reinforcing family members' exclusion and powerlessness. Cathy shared:

Even if there is only a door between us and him, it feels like there is a huge distance to him. (P02)

Family members perceived visitation restrictions as an established institutional norm, leaving them little choice but to adapt their daily routines, such as balancing work, childcare, and household responsibilities, to align with ICU schedules. As illustrated in a fieldnote:

Peter (P35) visited for about 10 min, leaving before visitation time ended. When I asked about his early departure, Peter explained that he was in a hurry to pick up his grandson from school at 4 p.m. (Fieldnote 36, 14 December 2021)

The policy allowing only one family member per patient further placed families in complex situations, requiring difficult negotiations about who should visit. This decision‐making process revealed internal hierarchies and obligations. For instance, some families prioritised visits from certain members due to perceived authority or representational roles, such as the eldest son. Simon explained:

I think it would be better to allow two family members to visit…one is the patient's husband or wife, and another is the patient's eldest son or other children […] because the eldest son in a family has a strong voice among siblings. (P26)

#### 5.1.2 Being Kept in the Dark

Most ICU admissions were unplanned, leaving family members unprepared for the life‐threatening nature of critical illness. The ICU entrance door became symbolic, representing a threshold beyond which lay a world grappling with danger, threats, and the potential for hope or miracles, as Anna described:

This entrance door is a life‐and‐death door…patients being admitted here are in really critical condition, and those with less severe conditions are not sent here. (P01)

All family members were aware that ICU admission signified a severe and life‐threatening condition. Hence, uncertainty was a constant emotional strain, as Cathy expressed:

When we see these several words (‘intensive care unit’), when my father‐in‐law was admitted here, it was like torture for the whole family […] We would feel reassured only when he is transferred out of the ICU. (P02)

To manage this uncertainty, family members sought opportunities to access both the patient and ICU staff, particularly physicians and nurses, who were seen as essential sources of information. However, family members highlighted the frustration of restricted access to communication channels, with their information needs unmet. Janet said:

There is only one opportunity to talk with physicians daily […] As family members, we want to know his condition continuously! (P10)

Some ‘intrusive’ situations, such as witnessing the death of other patients being transferred out of the ICU, further exacerbated family members' sense of powerlessness. These emotionally intense situations often led families to reflect on their own family member's condition, prompting them to seek continuous updates to reassure themselves about their family member's stability. Martin highlighted this:

A few days ago, a patient was being resuscitated inside. His family members were all standing at the entrance door, yelling and crying…I could see that they were all hoping the patient would pull through. As family members, the only thing we can do is wait for the information, whether good or bad. (P15)

To cope with restricted information access, some family members developed their own strategies to interpret patient conditions through learning situational cues. For instance, Dave described how the presence or absence of physicians signalled changes:

If physicians don't look for family members, it means everything is good with the patient, but if they look for you, it means something bad happened […] Physicians will not look for us if there is nothing happening inside. (P03)

Many family members also adopted the strategy of staying physically close to the ICU, which offered a sense of proximity and allowed them to respond quickly to any changes in the patient's condition. Janet explained:

Even if physicians say that they will inform us promptly if there are any changes, patients admitted to this unit are all in critical condition…if any changes happen, the fastest way is to stay right outside the ICU. (P10)

#### 5.1.3 Exclusion From Patient Care

The restricted visitation policy also meant family members were excluded from patient care, leaving their needs for assurance unmet. Many saw themselves as patient advocates, ensuring that the patients' needs were addressed. Cherry explained:

If one family member were allowed inside, it would benefit the patient… When my brother went to visit my mother, she said, ‘I feel thirsty. I feel thirsty.’ We believe nurses give her drinks, but…it may not always be dealt with immediately. (P31)

Family members of conscious patients particularly emphasised their role in providing emotional and psychological support, given that the ICU environment itself, characterised by intrusive treatment activities and unfamiliar staff, was a source of stress. In contrast, family presence, through familiar voices and touch, was perceived to have a consoling effect and provide a sense of security for patients. Grace reflected on this:

In the information communication with Grace (P21‐granddaughter), she said, ‘My grandma is inside on her own, and I feel she must be really helpless.’ Every time a nurse calls her, ‘Hey, madam, open your eyes,’ she doesn't even know who is calling her. She must feel more upset and agitated. (Fieldnote 19, 24 June 2021)

Rather than performing physical care, family members expressed their role in ‘being there,’ providing emotional support and companionship in ways that complemented the work of ICU staff. Kelly explained:

If one family member were allowed inside to accompany him and talk with him, he would not feel so lonely in the ICU. Nurses do their job—caring for the patient and giving medications—but having family there would be good for him, right? (P13)

Exclusion from patient care also influenced family members' perceptions of the quality of care. Some family members fostered a sense of mistrust due to a lack of direct observation. Many expressed a ‘seeing is believing’ mindset, as Paul noted:

I feel…the nursing aspect…is unsatisfactory […] But the fact is, you can't see how they actually care for the patient, right? It is closed here, so you can't really see it. (P19)

During visits, family members were often observed to closely inspect patients to assess care quality, checking for hygiene, skin conditions, and overall appearance. Anna described:

I touched her back to see whether there is sweat on her back or whether the skin is damaged…whether the skin is red or swollen, but they (nurses) may not notice it. (P01)

Additionally, the exclusion from patient care led to unfulfilled familial role responsibilities, with some family members feeling guilty about their limited involvement. For instance, Kelly described how her husband expressed frustration at her absence, reinforcing the perceived need for family presence:

Yesterday, I visited my husband […] He reproached me, saying, ‘Why haven't you come to see me these days? Where have you been?’ I know he was angry at me for not being with him, for leaving him alone here. (P13)

It was clear that the restricted visitation policy structurally positioned family members as outsiders, hindering their ability to meet their visitation needs. However, they navigated these challenges of exclusion by building supportive networks in the waiting area, as illustrated in the following section.

### 5.2 Being an Insider

#### 5.2.1 Receiving Support From Family Members

Keeping a vigil outside the ICU became an everyday routine for many families. The waiting area was often crowded with extended families and functioned as a temporary dwelling. Here, family members arranged seating and coordinated daily activities such as eating and sleeping. A fieldnote captured this scene:

This observation session took place in the waiting area, half an hour before the visitation time at 3 p.m. I found that there were no free seats. Several family members were standing and talking with each other. Several were sitting on the staircases. In the corner of the waiting area, blankets and quilts had been tied up and positioned there. (Fieldnote 06, 05 May 2021)

Although some participants mentioned physical discomfort due to limited seating, restricted access to food or bathroom facilities, all emphasised the importance of being there. Such presence was seen as fulfilling their cultural and familial responsibilities, rooted in the values of filial piety, which provided emotional relief. Oliver explained:

I stayed in the waiting area almost every day, except when I left for a meal. You can understand that there is a blood relation. Anyone in such a situation would behave in this way. (P29)

Waiting carried a significant emotional strain. Yet, collective family presence became an emotional resource to help endure the seemingly endless waiting. Cherry described:

I felt the solidarity between our siblings […] as children, none of us wanted to leave…We didn't want to go back, because staying in the waiting area made us feel close to mum. (P31)

The designated family member to visit the patient became the key conduit between the waiting area and the ICU. Their brief bedside time was used strategically to observe the patient's condition and gather updates from nurses or physicians. This information was then shared with the extended family, as Ken described:

My brother came to see my dad today […] He said he saw our dad had reactions when he called him at the bedside. We definitely felt some assurance (P11)

#### 5.2.2 Connecting With Other Patients' Families

For those who remained outside the ICU for extended periods, the waiting area was a communal space shared with families of other patients. Despite being strangers with no previous acquaintance, shared experiences fostered mutual understanding and emotional connection. Martin reflected:

Every family that has a family member inside would stay here. Who could feel reassured enough to go back home? Yesterday, I woke up at midnight, around 9 pm. I couldn't fall asleep until 12 p.m. I saw many families still awake. I thought to myself, ‘Well, just keep lying like this.’ (P15)

These connections were often underpinned by empathy and concern. While families exchanged information about their own ill family members, they were also mindful of others' emotional fragility, as Dave said:

We have some conversations with other families—‘How was your family member admitted to the ICU? How long has your family member been in the ICU?’ However, we are cautious about these discussions because all the patients inside are in critical condition, and their families are already distressed. Talking more about it could make things more distressing. (P03)

For many, the waiting area became a space of empowerment, where social connections created a sense of belonging. Jim reflected on how this communal space provided some comfort and eased distress:

Even if I kept myself staying at home now, I couldn't fall asleep as well. I can only stay here in the waiting area. It is noisy here, but it makes me feel better. (P34)

However, not all found reassurance in these interactions. Witnessing others' suffering, especially when bad news was delivered, could intensify emotional distress and trigger collective anxiety. Janet shared:

One family member was crying loudly after receiving bad news from the physicians. Her strong emotions almost made the other families cry because it reminded us of our own ill family member inside. (P10)

Some family members chose to maintain their own family boundaries, distancing themselves from other families. For example, they sat together with their own family members, minimised communication, or avoided eye contact. This was a coping strategy to preserve hope, as Leo described:

When we saw these bad scenes, we felt sorry for them, but we needed to maintain hope…We ask ourselves to only think about our own family members, not to think about others. (P12)

These findings illustrate how the restricted visitation policy intersected with culturally embedded family roles and relational dynamics within and between families, shaping family members' visitation experiences in the ICU.

## 6 Discussion

This focused ethnographic study provides a culturally sensitive, theoretically informed understanding of how the restricted visitation policy shaped family visitation in a Chinese adult ICU. Using Merton's (1972) conceptions of outsiders and insiders, the findings demonstrate that family experience was shaped by the interplay of structural constraints, culturally embedded family roles, and relational dynamics. These processes were spatially produced. Family members were structurally positioned as outsiders to the staff‐controlled ICU, while simultaneously constructing insider status in the socially meaningful family space of the waiting area.

The ICU's material configuration resembled what Newman (1972) calls ‘defensible space’, a spatial order that produced perceived zones of territorial control. The closed entrance door functioned both as a physical barrier and a symbolic marker of authority, reinforcing family members' perception of the ICU as staff territory. This spatial exclusion also led to an epistemic boundary, with limited access to information and restricted opportunities for involvement in care. Family members described emotional separation, heightened uncertainty, and powerlessness. Similar findings have been reported in other restricted contexts (Björk et al. 2019). By contrast, studies of more flexible policies show that relaxing visitation restrictions allows family members to maintain normality, have greater engagement with ICU staff, and build trust through direct observation of care practices (Mitchell and Aitken 2017; Wu et al. 2022). Consequently, family members reported higher satisfaction and reduced emotional distress (Rodriguez‐Ruiz et al. 2021).

In the Chinese cultural context, visitation was experienced as a moral obligation and a cultural expectation, rooted in filial piety and intergenerational solidarity. Our findings demonstrate that family members actively reclaimed presence through the waiting area. Here, waiting outside was not merely a passive state, but involved family members' agency of making meaning and negotiating relationships with one's own family and with other families, to navigate exclusion. The waiting area emerged as a socially and emotionally constructed space where families created a supportive community. Extended Chinese families provided an important support network, offering emotional, practical, and financial support. Shared experiences with other families often fostered a sense of belonging, consistent with Bournes and Mitchell's (2002) notion of ‘contentment with uplifting engagements’ amidst the turbulence of ICU waiting.

Our study extends this literature by revealing the dual nature of the waiting area. For some, it functioned as a space of empowerment, while for others, exposure to other families' crises intensified distress and prompted strategies to maintain family boundaries. This experience reflects previous work emphasising the centrality of the waiting environment to family experience and the need for comfort, privacy, and adequate space to empower families to meet their proximity needs during critical illness (Kynoch et al. 2021; Mitchell and Aitken 2017).

These findings call for critical reflection on prolonged restricted visitation policies and a shift toward flexible approaches that better accommodate family needs and cultural values. Importantly, the study advocates for context‐sensitive flexibility, meaning that visitation policies should be adapted to cultural, institutional, and practical conditions. In contexts like China, where extended family structures and culturally embedded caregiving obligations predominate, family members may prefer more frequent visits or the presence of multiple members at a time. These preferences should be carefully balanced against operational needs, such as workflow continuity of ICU staff and adequate space for clinical activities (Bailey et al. 2022). Rather than prescribing a one‐size‐fits‐all visitation model, we recommend a flexible policy framework that enables family members and ICU staff to negotiate arrangements that support mutual needs.

### 6.1 Strengths and Limitations of the Work

The strengths of the study lie in its rigorous methodological approach to data collection and analysis. Triangulation of multiple data sources facilitated a more comprehensive exploration of the research questions and enhanced the validity of the findings. The inductive and abductive analysis, informed by theoretical concepts of outsider and insider (Merton 1972), provided in‐depth interpretations of family visitation under the restricted visitation policy. These theoretically informed insights hold relevance beyond the immediate study context, potentially transferable to other settings with similarly prolonged restricted visitation policies.

However, this study had some limitations. As it was conducted in a single Chinese adult ICU, the findings are context‐specific to this particular setting. The study took place during the COVID‐19 pandemic, when heightened restrictions, such as limiting visitors to one at a time, were imposed. While the primary focus was on the prolonged 30‐min visitation policy, these additional pandemic‐specific restrictions may have influenced family members' perceptions, making the findings potentially time‐specific.

### 6.2 Recommendations for Further Research

Future research could examine the transferability of the theoretical concepts of outsider‐insider dynamics and a spatial perspective to other cultural and institutional contexts to explore family members' visitation experiences. This is particularly important in the post‐COVID‐19 era, as many ICUs worldwide continue to implement restricted visitation policies or delays in re‐involving family members (Dragoi et al. 2022). Additionally, future research could focus on diverse ethnic and cultural groups, enabling a more nuanced understanding of the interplay among cultural values, familial roles, and institutional practices related to ICU visitation.

### 6.3 Implications for Policy and Practice

Shifting long‐standing restricted visitation policies is undoubtedly complex, which requires sustained efforts and commitment. Based on the study findings, three key implications for policy and practice are proposed. First, ICU staff, particularly nurses, who have the most frequent interactions with family members at the bedside, need to be aware of how restricted visitation policies shape family members' experiences and perceptions. Reflexive group discussions, drawing on successful examples of policy transitions in other settings, may encourage staff to critically reconsider the assumption that restricted policies are the default option. Second, the study suggests that flexible visitation should be context‐sensitive, carefully balancing family members' cultural expectations and caregiving roles with the operational demands of ICU staff. Third, the development or revision of ICU visitation policies should involve a co‐design process, in which the perspectives of ICU staff, family members, and patients are incorporated to ensure policies are both culturally responsive and operationally feasible.

## 7 Conclusion

This study highlights the prolonged restricted visitation policy as a structural mechanism reinforcing family members' powerlessness by constructing the ICU as the territory of ICU staff and positioning family members as outsiders. In response, family members reclaimed presence in the waiting area, transforming it into a supportive community where they built connections within their own families and with other patients' families. These findings highlight the need to reconsider restricted visitation policies, particularly in culturally specific contexts such as China, where familial obligations are deeply embedded. By illuminating the intersection of structural constraints, cultural expectations, and relational dynamics, this study contributes to the global discourse on developing flexible visitation policies that are context‐sensitive to facilitate more meaningful family involvement.

## Ethics Statement

We confirm that any data utilised in the submitted manuscript has been lawfully acquired in accordance with the Nagoya Protocol on Access to Genetic Resources and the Fair and Equitable Sharing of Benefits Arising from their Utilisation to the Convention on Biological Diversity. This study was approved by the Research Ethics Committee of the School of Health in Social Science, The University of Edinburgh (Reference No. NURS059). Institutional approval was granted by the hospital and ICU through an official permission letter prior to the fieldwork. This letter confirmed permission to conduct the study within the hospital's ICU. Participation was voluntary, and all participants provided written informed consent prior to enrollment in the study.

## Conflicts of Interest

The authors declare no conflicts of interest.

## Supporting information


**File S1:** Interview guide for family members.

**Data S2:** Standards for Reporting Qualitative Research (SRQR)*.

## Data Availability

The data that support the findings of this study are available from the corresponding author upon reasonable request. The data are not publicly available due to privacy and ethical restrictions.
