# Supplementary material for: ‘Being an Outsider’ and ‘Being an Insider’: A Focused Ethnography of Family Members' Visitation Experiences in an Adult ICU
Source: J Adv Nurs. 2025 Nov 3;82(7):7441–52. doi: 10.1111/jan.70340 (PMC13267465; doi:10.1111/jan.70340)
Supplement: Supplementary file 1 — File S1: Interview guide for family members. [file JAN-82-7441-s002.docx]

**Supplementary File 1: Interview guide for family members**

| **Background information**  Can you tell me about how your family member was admitted to the ICU? |
| --- |
| How long has your family member been in the ICU? |
| How is your family member doing? |
| Have you had any prior experience with a family member being admitted to an ICU? |
| **Family members’ knowledge of the ICU visiting policy**  Can you describe the unit’s visiting policy? |
| How do you learn about the visiting policy? |
| **Experience with ICU visiting policy and practice**  What do you usually do when you visit your family member in the ICU?   - Follow-up questions based on observed behaviours of family members to elicit more explanations, e.g., checking the patient’s hygiene or engaging with staff.   Can you describe how you felt when visiting your family member in the ICU?  Can you share your experience of interacting with nurses in the ICU? |
| **Waiting experience**  Can you describe your feelings when waiting outside the ICU?   - Follow-up questions: How do you take care of yourself and other family members while your family member is in the ICU?   What do you usually do outside the ICU?   - Follow-up questions: What support have you received from other family members during this time? - Can you please describe this experience of interacting with other patients’ families while waiting outside? |
| **Perceptions of the ICU visiting policy and practice**  How do you perceive the current visiting time restrictions? |
| What changes, if any, do you think could be made to improve the current visiting policy? |
| ***Note***. The sequence of questions may be adjusted based on participants’ responses. Family members will be encouraged to share their thoughts on any aspect of the visiting policy and practices. Probing questions, such as *“Could you give an example?”*, *“Do you have any additional comments?”* will be used to elicit richer and more detailed responses. |
